# Bilateral Serous Detachment Associated with Latanoprost/Timolol Fixed Combination Use: A Report of One Phakic Case

**DOI:** 10.1155/2012/305379

**Published:** 2012-12-13

**Authors:** Esra Ayhan Tuzcu, Ugurcan Keskin, Mesut Coskun, Ozgur Ilhan, Mutlu Daglıoglu, Huseyin Oksuz

**Affiliations:** Faculty of Medicine, Mustafa Kemal University, 31200 Hatay, Turkey

## Abstract

We present a case of bilateral diffuse serous detachment associated with latanoprost/timolol fixed combination treatment which was recovered after changing treatment.

## 1. Introduction

 Latanoprost is the first prostaglandin *F*_2*α*_ analog in the world used for decreasing intraocular pressure which acts by increasing uveoscleral outflow of aqueous humour [1]. Timolol is a *β* adrenergic antagonist antiglaucomatous agent acting by decreasing aqueous humour production [2]. The most well-known local side effects of timolol and latanoprost are conjunctival hyperemia and ocular discomfort. Eyelid discoloration, iris pigmentation, increase in the length, number, color, and thickness of eyelashes, anterior chamber inflammation, cystoid macular edema, and corneal changes are the local side effects of latanoprost and the other prostaglandin analoges [3]. In this paper, we present a bilateral serous detachment in the macula associated with latanoprost/timolol fixed combination use. To the best of our knowledge no report about serous detachment associated with latanoprost/timolol fixed combination has been published before. 

## 2. Case Report

**Figure 1:**
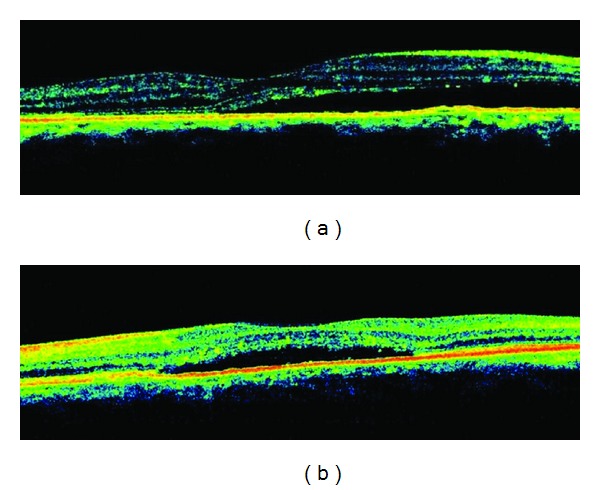
(a) Serous retinal detachment associated with latanoprost/timolol fixed combination use right eye view of the OCT. (b) Serous retinal detachment associated with latanoprost/timolol fixed combination use left eye view of the OCT.

**Figure 2:**
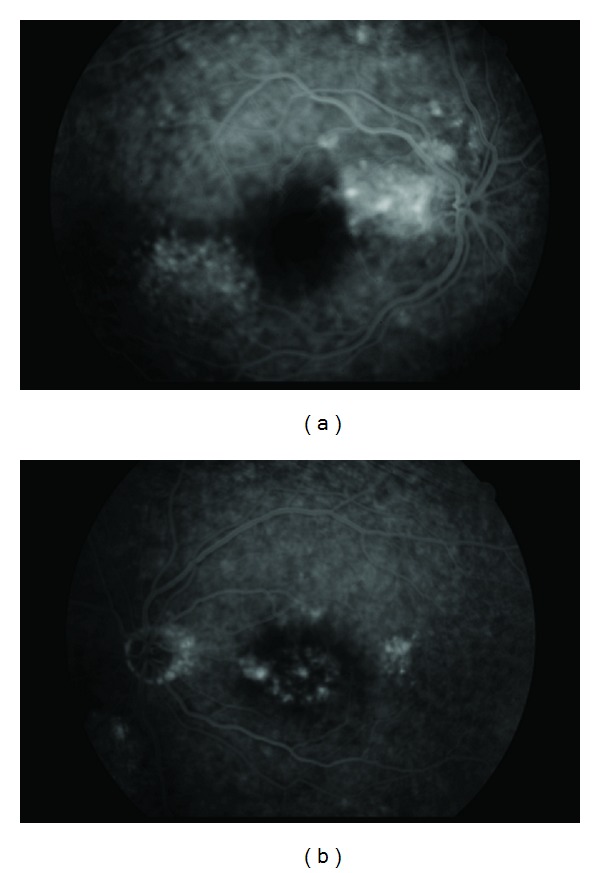
(a) Focal leakages in the macular region of the right eye view of the FFA. (b) Focal leakages in the macular region of the left eye view of the FFA.

52-year-old female patient with primary open-angle glaucoma using brinzolamide 1% twice daily and latanoprost/timolol maleat once daily admitted to our clinic with bilateral decrease in vision. In her examination, visual acuity was 0.2 in right eye and 0.6 in left eye and intraocular pressure was 15 mmHg in right eye and 13 mmHg in left eye. Her fundus examination revealed bilateral serous detachment. In OCT, macular thickness was 311 *μ*m in right eye (Figure 1(a)), 272 *μ*m in left eye (Figure 1(b)). There was no past history of uveitis, ocular surgery, and systemic disease. Fluorescein fundus angiogram (FFA) showed focal leakages in the macular region of the right and left eyes (Figures 2(a) and 2(b)).

We considered the serous retinal detachment as a side effect of latanoprost, and we changed latanoprost/timolol fixed combination with brimonidine/timolol fixed combination. After 1 month of changing treatment, visual acuity was increased to 0.6 in right eye and 0.8 in left eye, and intraocular pressure was measured as 12 mmHg in right eye and 13 mmHg in left eye. OCT macular thickness was decreased to 215 *μ*m in right eye and 223 *μ*m in left eye. 

## 3. Discussion

In this paper we describe a phakic case with bilateral serous retinal detachment of the macula with latanoprost/timolol fixed combination. Visual acuity of patient was increased dramatically after cessation of latanoprost/timolol fixed combination, and a significant decrease in macular thickness was observed in OCT. These findings made us think about the relationship between latanoprost/timolol fixed combination and serous retinal detachment. There are many causes of serous retinal detachment: inflammatory, infectious, age-related macular degeneration, optic nerve, vascular, and neoplastic disorders [4]. In our patient's absence of past history of these disorders, bilateral serous retinal detachment was developed during latanoprost/timolol fixed combination treatment. Ozkan and Karabaş reported similar case of a 67-year-old woman who developed serous detachment with association latanoprost use. The patient was phakic in one eye [4]. Sauer et al. reported increased myopia of a 59-year-old man who developed serous detachment with association latanoprost use [5]. The patient was phakic having increased myopia in both eyes. Pathogenesis of central serous chorioretinopathy (CSCR) is unclear. Haimovici et al. showed that inflammatory and autoimmune conditions might be involved to CSCR [6]. Camras and Miyake claimed that endogenous prostaglandins were induced by latanoprost [7]. 

Cystoid macular edema is one of the well-known and infrequent side effects of prostaglandin analogs. The pathogenesis of the serous detachment in our patient may be the same with the pathogenesis of cystoid macular edema. Serous detachment associated with latanoprost may be caused by latanoprost directly or by the induced prostaglandins, and further studies are needed to clarify this point. In conclusion, in glaucoma and ocular hypertension, prostaglandin analogues must not be used in high-risk patients, and caution is needed in all patients for development of macular edema and serous detachment.
